# Systemic immune-inflammation Index and blood pressure control are independent and interactive predictors of atrial fibrillation recurrence after ablation

**DOI:** 10.3389/fcvm.2026.1656903

**Published:** 2026-05-15

**Authors:** Yuhang Wei, Chenxi Cao, Yunzhe Wang, Pengli Yang, Wenjing Zhang, Jiahong Shangguan, Xijia Wang, Jiaqi Gao, Wenzhe Zong, Gangqiong Liu

**Affiliations:** 1 Department of Cardiology, Key Laboratory of Cardiac Injury and Repair of Henan Province, First Affiliated Hospital of Zhengzhou University, Zhengzhou, China; 2 Department of Cardiology, Beijing Anzhen Hospital of Capital Medical University and Beijing Institute of Heart Lung and Blood Vessel Diseases, Beijing, China

**Keywords:** atrial fibrillation, blood pressure control, catheter ablation, hypertension, recurrence, systemic immune-inflammation index

## Abstract

**Background:**

Atrial fibrillation (AF) recurrence remains a major limitation after radiofrequency catheter ablation (RFCA), especially in patients with hypertension. Systemic immune-inflammation index (SII) has emerged as a novel marker of systemic inflammation, yet its prognostic value in this population is unclear.

**Objective:**

To assess the predictive value of pre-ablation SII for AF recurrence and explore the modifying effect of blood pressure (BP) control status in hypertensive patients.

**Methods:**

In this retrospective cohort study, 568 hypertensive AF patients undergoing first-time RFCA were enrolled. Patients were stratified by SII quartiles and BP control status. Cox regression, ROC analysis, and restricted cubic spline modeling were used to evaluate associations with AF recurrence.

**Results:**

During a mean 34-month follow-up, 17.3% experienced AF recurrence. Elevated SII was independently associated with recurrence risk (HR per 100-unit increase: 1.138; *P* < 0.001). Patients with both high SII and poorly controlled BP had a > 4-fold increased risk (HR: 4.061). SII outperformed conventional markers and significantly improved model discrimination (C-statistic: 0.755).

**Conclusion:**

Pre-ablation SII is an independent and superior predictor of AF recurrence after RFCA in hypertensive patients, particularly when BP is poorly controlled. Combined assessment of SII and BP control may improve individualized risk stratification.

## GRAPHICAL ABSTRACT

**Figure:**
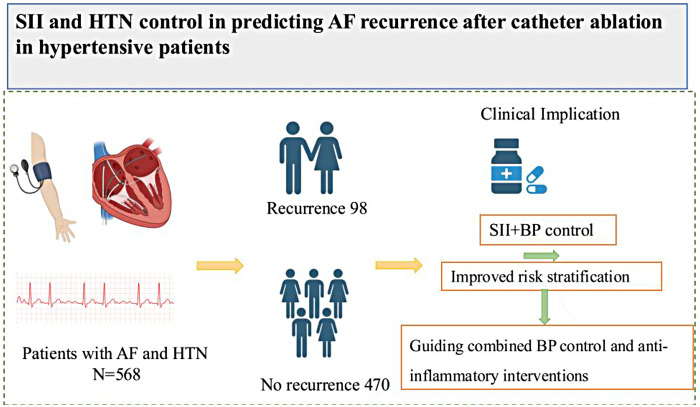

## Highlights

- This is the first study to systematically evaluate the independent predictive value of the systemic immune-inflammation index (SII) for atrial fibrillation (AF) recurrence after catheter ablation specifically in hypertensive patients.
- It identifies blood pressure control status as a significant effect modifier of the SII–AF recurrence association.
- The study establishes a a pronounced clinical synergy between elevated SII and poor blood pressure control, highlighting a high-risk subgroup characterized by a compounded fourfold increase in recurrence risk (HR = 4.061).
- SII demonstrated superior predictive performance compared to conventional markers such as AF duration and left atrial volume, underscoring its potential utility in clinical risk stratification.

## 1 Introduction

Atrial fibrillation (AF) is one of the most common arrhythmias encountered in clinical practice, affecting over 33 million individuals globally. Its pathogenesis is closely linked to inflammation, oxidative stress, and structural remodeling of the atria (1). Hypertension (HTN) is a major risk factor for AF, with hypertensive individuals having a 73% higher risk of developing AF compared to normotensive counterparts (2). Moreover, more than 70% of patients with AF have comorbid hypertension (3). Radiofrequency catheter ablation (RFCA) is a widely adopted treatment strategy and is a Class I recommendation for patients with paroxysmal AF who are refractory to or intolerant of antiarrhythmic drug therapy (4). Despite its efficacy, post-ablation AF recurrence remains a significant clinical challenge, particularly in patients with coexisting HTN, where hypertension-induced cardiac structural and electrical remodeling may exacerbate the risk of recurrence and complicate treatment (5, 6). Therefore, early identification and management of recurrence risk factors is essential to optimize ablation outcomes.

In recent years, the SII—a composite marker derived from neutrophil, platelet, and lymphocyte counts—has gained attention for its ability to reflect systemic immune-inflammatory dysregulation (7). Elevated SII has been associated with atherosclerosis, coronary artery disease, and AF (8). Mechanistically, neutrophils contribute to atrial injury via the release of myeloperoxidase and neutrophil extracellular traps (NETs) (9), while platelet-derived particles promote fibroblast proliferation through platelet-derived growth factor (PDGF) and transforming growth factor-β (TGF-β) signaling (10). Concurrently, lymphopenia may indicate impaired regulatory T cell (Treg) activity, facilitating uncontrolled Th17 cell infiltration and interleukin-17 (IL-17)-mediated atrial fibrosis (11) While SII has shown promise in predicting AF recurrence, prior studies have seldom accounted for BP control status (12).

Accordingly, this study aimed to evaluate the prognostic value of pre-ablation SII for AF recurrence in hypertensive patients undergoing RFCA and to explore how BP control status may modify this association. By integrating inflammation and hemodynamic status, this investigation seeks to provide new insights into individualized risk stratification and targeted intervention in this high-risk population.

## 2 Methods

### 2.1 Study design and populations

We conducted a single-center, retrospective observational cohort study involving 630 patients with AF and comorbid HTN who underwent first-time RFCA during hospitalization in the Department of Cardiovascular Medicine at the First Affiliated Hospital of Zhengzhou University between January 2020 and December 2022. All patients received RFCA for the first time. The exclusion criteria were as follows: (1) previous left atrial ablation or cardiac surgery; (2) significant structural heart disease (e.g., congenital heart disease or valvular heart disease); (3) secondary AF (e.g., due to hyperthyroidism or pulmonary embolism); (4) active infection, known hematological disorders, autoimmune diseases, malignancy, or severe hepatic or renal dysfunction; and (5) incomplete clinical data.

In total, 62 patients were excluded due to incomplete clinical data or loss to follow-up, resulting in the consecutive enrollment of 568 eligible patients. The study was conducted in accordance with the principles of the Declaration of Helsinki, and the study protocol was approved by the Ethics Committee of the First Affiliated Hospital of Zhengzhou University. Follow-up data were obtained through systematic review of medical records and telephone interviews.

### 2.2 Data collection and definitions

We used standardized spreadsheets to collect retrospective clinical data. Baseline demographic and clinical characteristics included sex, age, body mass index (BMI), atrial fibrillation (AF) type (paroxysmal or persistent), duration of AF history, smoking status (current smoker), alcohol consumption, history of coronary artery disease (CAD), diabetes mellitus, and transient ischemic attack (TIA).

Venous blood samples were collected during hospitalization after at least 8 h of fasting. Laboratory parameters included red blood cell count (RBC), platelet count (PLTC), neutrophil count (NEU), lymphocyte count (LYM), albumin (Alb), estimated glomerular filtration rate (eGFR), creatinine (Cr), blood uric acid (BUA), triglycerides (TG), high-density lipoprotein cholesterol (HDL-C), low-density lipoprotein cholesterol (LDL-C), and other routine indices. Echocardiographic data were obtained from the first transthoracic echocardiography performed upon admission, including left ventricular diameter (LVD), left atrial diameter (LAD), and left ventricular ejection fraction (LVEF). Left atrial volume was derived from left atrial–pulmonary vein computed tomography angiography.

The systemic immune-inflammation index (SII) was calculated as: platelet count × (neutrophil count/lymphocyte count). The CHA₂DS₂-VASc score was computed based on hospital admission data, incorporating the presence of congestive heart failure, hypertension, age ≥ 75 years, diabetes mellitus, prior stroke, transient ischemic attack (TIA), thromboembolism, vascular disease, age 65–74 years, and female sex.

Hypertension was defined as systolic BP ≥ 140 mmHg, diastolic BP ≥ 90 mmHg, or the current use of antihypertensive medications. Uncontrolled hypertension (Poor BP control) was specifically defined as persistently elevated BP despite treatment with three or more antihypertensive agents. To ensure diagnostic accuracy and minimize measurement bias, persistence was determined via ambulatory blood pressure monitoring (ABPM) or home blood pressure monitoring (HBPM), with thresholds set at a mean systolic BP ≥ 130 mmHg and/or diastolic BP ≥ 80 mmHg during the 3-month post-ablation follow-up period.

Paroxysmal AF was defined as AF episodes terminating spontaneously or with intervention within 7 days, typically lasting less than 48 h. Persistent AF was defined as AF episodes lasting longer than 7 days. The duration of AF was calculated from the time of symptom onset or first diagnosis to the date of RFCA. Body mass index (BMI) was calculated as weight in kilograms divided by the square of height in meters (kg/m²).

### 2.3 Ablation strategy

All ablation procedures were guided by the CARTO3 three-dimensional electro-anatomical mapping system (Biosense-Webster, Diamond Bar, CA, USA) and were tailored according to AF type. Patients with paroxysmal AF underwent circumferential pulmonary vein isolation (CPVI), while those with persistent AF received CPVI combined with linear ablation of the left atrial roof, mitral isthmus, and tricuspid isthmus. Ablation targets were selected primarily based on anatomical landmarks, and routine substrate modification of low-voltage areas was not performed as a first-line approach. If non-pulmonary vein triggers or atrial flutter circuits were identified during the procedure, targeted ablation was performed based on electrophysiological induction results. Procedural endpoints included complete electrical isolation of all pulmonary veins, bidirectional conduction block across additional ablation lines, and restoration of sinus rhythm via synchronized direct-current cardioversion (100–200 J) if atrial arrhythmias persisted post-ablation. All patients underwent a routine 30 min observation period following the procedure to assess immediate rhythm stability and procedural efficacy.

### 2.4 Postoperative management and follow-up

All patients received antiarrhythmic drug (AAD) therapy for at least 3 months following ablation to prevent early recurrence. Subsequent medication adjustments were made collaboratively based on physician assessment and patient preference. Scheduled follow-up visits were conducted at 1-, 3-, and 6-months post-procedure, during which patients underwent 12-lead electrocardiography (ECG) and 24 h Holter monitoring. Beyond 6 months, follow-up was continued either in person or remotely. Additional ECG or Holter recordings were obtained whenever patients reported symptoms suggestive of AF. Recurrence was defined as any documented atrial tachyarrhythmia lasting ≥30 s on ECG or Holter monitoring occurring after the completion of this period. Any arrhythmic episodes occurring within the initial 3 months were not counted as primary endpoint events, and these patients were neither excluded from the study nor censored at that time; instead, they remained in the cohort for long-term survival analysis starting from the end of the blanking period. The median follow-up duration was 34 months.

### 2.5 Statistical analysis

Statistical analyses were conducted using a combination of R software (version 4.3.1; R Foundation for Statistical Computing, Vienna, Austria) and SPSS (version 29.0; IBM Corp., Armonk, NY, USA). Continuous variables were expressed as mean ± standard deviation, while categorical variables were presented as counts and proportions. Patients were stratified into four groups according to quartile thresholds for baseline SII, calculated using Tukey's hinge method. The Kolmogorov–Smirnov test was employed to evaluate the normality of continuous variables, and appropriate parametric or non-parametric tests were applied accordingly to compare intergroup differences.

To explore the association between SII, blood pressure control status, and AF recurrence, a multivariable Cox proportional hazards model was constructed. The predictive performance of SII for post-ablation AF recurrence was evaluated using receiver operating characteristic (ROC) curve analysis and the area under the curve (AUC). Kaplan–Meier curves were generated to estimate recurrence-free survival, and group differences were assessed using the log-rank test. The dose-response relationship between baseline SII and recurrence risk was modeled using restricted cubic spline (RCS) regression. To assess the incremental predictive value of SII, DeLong test was used to compare AUCs between models. Additionally, Net Reclassification Improvement (NRI) and Integrated Discrimination Improvement (IDI) metrics were calculated to quantify improvements in model discrimination. A two-tailed *p*-value <0.05 was considered statistically significant for all analyses.

## 3 Results

### 3.1 Clinical characteristics of patients with AF

**Figure 1:**
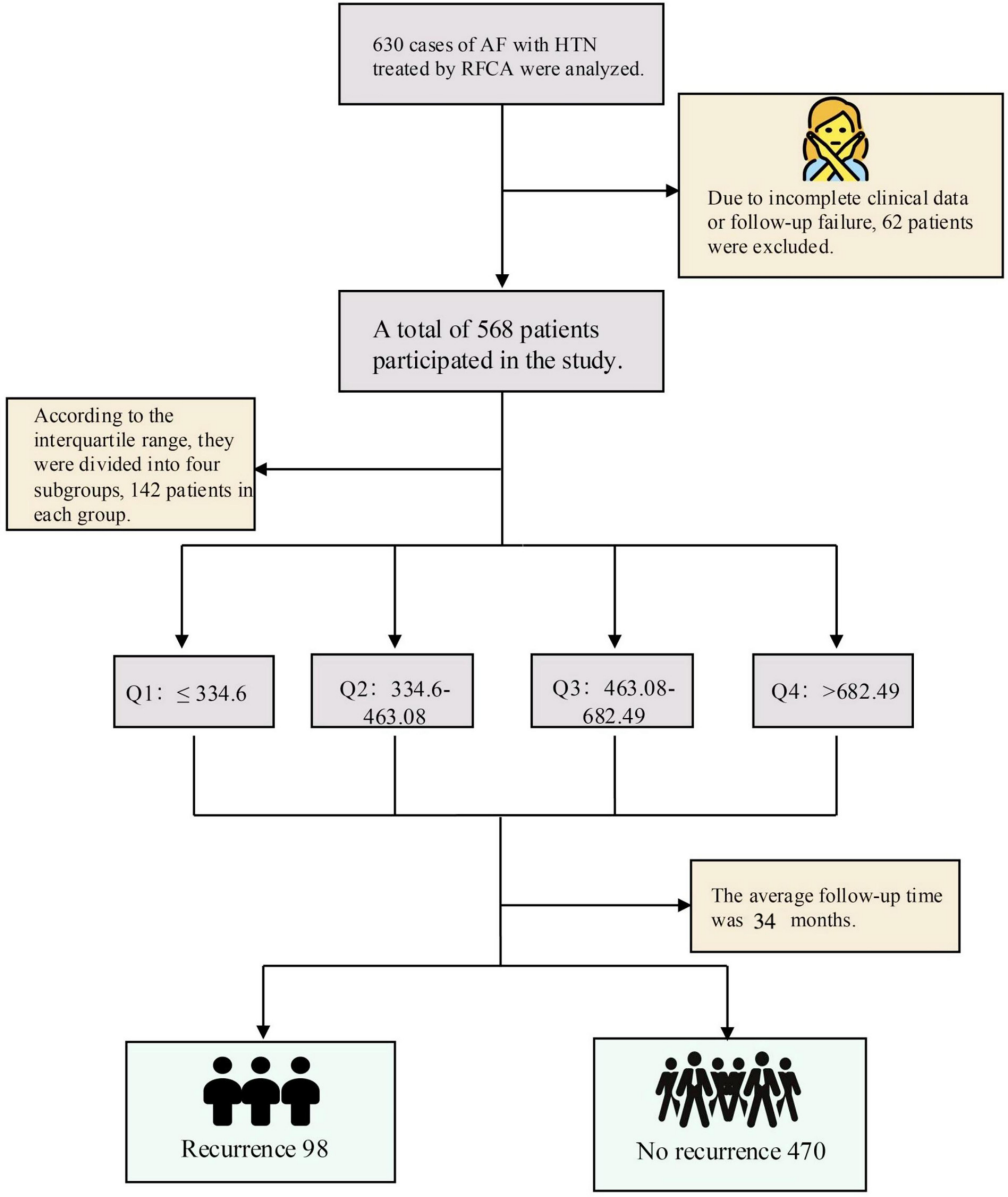
AF, atrial fibrillation; HTN, hypertension; RFCA, radiofrequency catheter ablation; SII, systemic immune-inflammation Index. Patients were stratified into quartiles based on baseline SII values: Q1 (≤334.6), Q2 (334.6–463.08), Q3 (463.08–682.49), and Q4 (>682.49).

A total of 630 patients diagnosed with atrial fibrillation (AF) and concomitant hypertension (HTN) who underwent successful radiofrequency catheter ablation (RFCA) were initially screened for this study. After excluding individuals with incomplete clinical data or lost to follow-up, 568 patients were ultimately included in the final analysis (Figure 1). To investigate the potential relationship between systemic immune-inflammation index (SII) and clinical outcomes, patients were categorized into four groups based on SII quartiles (Q1–Q4), with the lowest quartile (Q1) serving as the reference group for subsequent comparisons.

**Figure 2:**
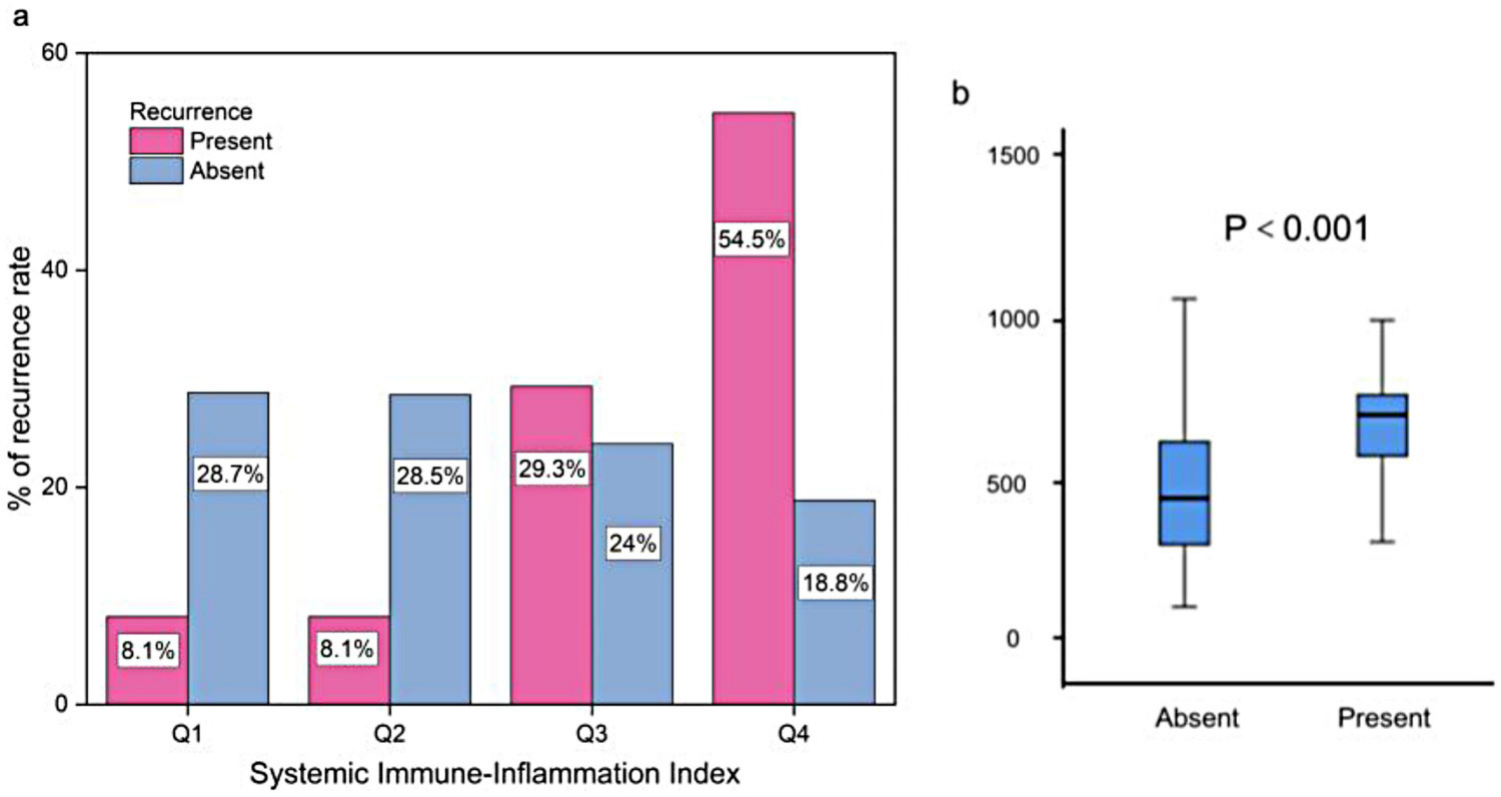
**(A)**. Percentage of the patients developing post-ablation AF recurrence stratified by quartile **(Q)** of the SII. **(B)**. Preoperative SII values were compared between patients who experienced AF recurrence and those who did not, *P* < 0.001.

During the follow-up period, 98 patients (17.3%) experienced recurrence of AF. Baseline clinical characteristics of patients with and without recurrence are summarized in Table 1. Compared with the non-recurrence group, those who experienced AF recurrence were more likely to be female, had a longer history of AF, higher CHA₂DS₂-VASc scores, increased left atrial volume, and elevated platelet count, neutrophil count, and SII. Recurrence rates also increased progressively across SII quartiles, and patients with recurrence showed significantly higher preoperative SII levels (Figure 2). Additionally, a greater proportion of patients in the recurrence group had poorly controlled HTN (all *P* < 0.05). No statistically significant differences were found between the two groups in terms of age, BMI, smoking and alcohol consumption, DM, or lipid profiles including TG, LDL-C, and HDL-C (all *P* > 0.05).

**Table 1:** Baseline characteristics of the study population.

Variables	AF recurrence (−) (*N* = 470)	AF recurrence (+) (*N* = 98)	*P*
Clinical characteristics			
Female, *n* (%)	183 (38.9%)	50 (51.0%)	0.036*
Age (years)	62.3 ± 9.33	63.6 ± 9.28	0.200
BMI (kg/m^2^)	25.8 ± 3.36	26.3 ± 3.29	0.106
AF type			
Paroxysmal, *n* (%)	243 (51.7%)	41 (41.8%)	0.096
Persistent, *n* (%)	227 (48.3%)	57 (58.2%)	
Duration of AF history(months)	12 (2.0, 36.0)	13 (6.0, 74.5)	0.002*
Current smoker, *n* (%)	125 (26.6%)	19 (19.4%)	0.172
Alcohol intake, *n* (%)	112 (23.8%)	18 (18.4%)	0.299
History of CAD, *n* (%)	179 (38.1%)	39 (39.8%)	0.839
Diabetes mellitus, *n* (%)	104 (22.1%)	29 (29.6%)	0.145
TIA, *n* (%)	106 (22.6%)	30 (30.6%)	0.116
CHA2DS2-VASc score	3.30 ± 1.65	3.63 ± 1.60	0.026*
Echocardiographic			
Left ventricle diameter (mm)	47.4 ± 4.83	47.5 ± 5.02	0.960
Left atrial diameter (mm)	40.2 ± 6.34	41.1 ± 5.94	0.109
LVEF (%)	60.7 ± 6.64	60.2 ± 7.84	0.807
Left atrial volume(mm³)	155 ± 47.8	165 ± 45.2	0.028*
Laboratory test			
RBC(×10^12^/L)	4.45 ± 0.510	4.38 ± 0.593	0.313
PLT(×10^9^/L)	201.79 ± 52.49	245.89 ± 42.93	<0.001*
NEU(×10^9^/L)	3.89 ± 1.34	4.39 ± 1.21	<0.001*
LYM(×10^9^/L)	1.75 ± 0.491	1.68 ± 0.385	0.242
Alb(g/L)	41.5 ± 3.64	41.4 ± 3.74	0.721
TG (mmol/L)	1.47 ± 0.866	1.69 ± 2.08	0.973
LDL-C (mmol/L)	2.07 ± 0.76	2.13 ± 0.8	0.512
HDL-C(mmol/L)	1.08 ± 0.258	1.07 ± 0.286	0.457
eGFR(mL/min/1.73m^2^)	86.3 ± 16.4	84.6 ± 18.3	0.648
Cr(*μ*mol/L)	76.0 ± 21.0	76.7 ± 33.0	0.240
BUA(μmol/L)	328 ± 93.0	322 ± 112	0.518
SII	491 ± 287	665 ± 211	<0.001*
SII_Quartiles			
Q1	135 (28.7%)	7 (7.1%)	<0.001*
Q2	134 (28.5%)	8 (8.2%)	
Q3	113 (24.0%)	29 (29.6%)	
Q4	88 (18.7%)	54 (55.1%)	
Follow-up parameters			
Follow-up time (months)	33.9 ± 9.65	17.4 ± 11.8	<0.001*
Hypertension control statusesn (%)			
Bad blood pressure control, *n* (%)	176 (37.4%)	62 (63.3%)	<0.001*
Well blood pressure control, *n* (%)	294 (62.6%)	36 (36.7%)	

AF, atrial fibrillation; Alb, albumin; BMI, body mass index; BUA, blood uric acid; CAD, coronary artery disease; Cr, creatinine; eGFR, estimated glomerular filtration rate; HDL-C, high-density lipoprotein cholesterol; LDL-C, low-density lipoprotein cholesterol; LYM, lymphocyte; LVEF, left ventricular ejection fraction; NEU, neutrophil; PLT, platelet; RBC, red blood cell; SII, systemic Immune-Inflammation Index; TG, triglycerides; TIA, Transient Ischemic Attack.

*P < 0.05.

Based on the degree of BP control observed during postoperative follow-up, patients were categorized into a good BP control group (*n* = 330) and a poor BP control group (*n* = 238). Detailed baseline characteristics of the two groups are presented in Table 2. The poor BP control group exhibited a significantly higher recurrence rate of AF, along with a longer AF history, increased prevalence of CAD and DM, and elevated CHA₂DS₂-VASc scores (all *P* < 0.05). In addition, this group demonstrated a lower eGFR. In contrast, no statistically significant differences were noted between the groups with respect to sex, age, BMI, echocardiographic structural parameters, or most laboratory indices.

**Table 2:** Baseline characteristics of patients stratified by hypertension control Status.

Variables	Bad blood pressure control, *n* (*N* = 238)	Well blood pressure control (*N* = 330)	*P*
Clinical characteristics			
Female, *n* (%)	103 (43.3%)	130 (39.4%)	0.400
Age (years)	63.4 ± 9.35	61.9 ± 9.27	0.052
BMI (kg/m2)	26.1 ± 3.37	25.8 ± 3.33	0.367
Atrial fibrillation type			
Paroxysmal, *n* (%)	121 (50.8%)	163 (49.4%)	0.799
Persistent, *n* (%)	117 (49.2%)	167 (50.6%)	
Duration of AF history(months)	13 (4.0, 48.0)	12 (2.0, 36.0)	0.015*
Current smoker, *n* (%)	54 (22.7%)	90 (27.3%)	0.254
Alcohol intake, *n* (%)	48 (20.2%)	82 (24.8%)	0.227
History of CAD, *n* (%)	106 (44.5%)	112 (33.9%)	0.013*
Diabetes mellitus, *n* (%)	67 (28.2%)	66 (20.0%)	0.031*
TIA, *n* (%)	61 (25.6%)	75 (22.7%)	0.484
CHA2DS2-VASc score	3.54 ± 1.70	3.23 ± 1.59	0.019*
Echocardiographic			
Left ventricle diameter (mm)	47.7 ± 4.88	47.3 ± 4.84	0.580
Left atrial diameter (mm)	40.4 ± 6.22	40.3 ± 6.32	0.928
LVEF (%)	60.4 ± 7.46	60.8 ± 6.40	0.636
Left atrial volume(mm3)	162 ± 48.4	153 ± 46.6	0.05
Laboratory test			
RBC(×1012/L)	4.41 ± 0.515	4.46 ± 0.533	0.406
PLT(×109/L)	207.09 ± 53.36	212.60 ± 53.86	0.227
NEU(×109/L)	4.02 ± 1.27	3.95 ± 1.38	0.409
LYM(×109/L)	1.71 ± 0.476	1.75 ± 0.473	0.179
Alb(g/L)	41.3 ± 3.81	41.7 ± 3.53	0.298
TG (mmol/L)	1.55 ± 1.50	1.48 ± 0.855	0.992
LDL (mmol/L)	2.06 ± 0.79	2.10 ± 0.76	0.638
HDL (mmol/L)	1.05 ± 0.267	1.09 ± 0.259	0.212
eGFR(mL/min/1.73m2)	84.2 ± 17.1	87.3 ± 16.5	0.036*
Cr(μmol/L)	77.5 ± 25.8	75.1 ± 21.6	0.363
BUA(μmol/L)	332 ± 103	324 ± 90.9	0.583
SII	534 ± 259	511 ± 299	0.085
Follow-up parameters			
Recurrence, *n* (%)			
No	176 (73.9%)	294 (89.1%)	<0.001*
Yes	62 (26.1%)	36 (10.9%)	
Follow-up time (months)	29.7 ± 12.7	32.1 ± 11.1	0.060

AF, atrial fibrillation; Alb, albumin; BMI, body mass index; BUA, blood uric acid; CAD, coronary artery disease; Cr, creatinine; eGFR, estimated glomerular filtration rate; HDL-C, high-density lipoprotein cholesterol; LDL-C, low-density lipoprotein cholesterol; LYM, lymphocyte; LVEF, left ventricular ejection fraction; NEU, neutrophil; PLT, platelet; RBC, red blood cell; SII, systemic Immune-Inflammation Index; TG, triglycerides; TIA, Transient Ischemic Attack.

*P < 0.05.

**Figure 3:**
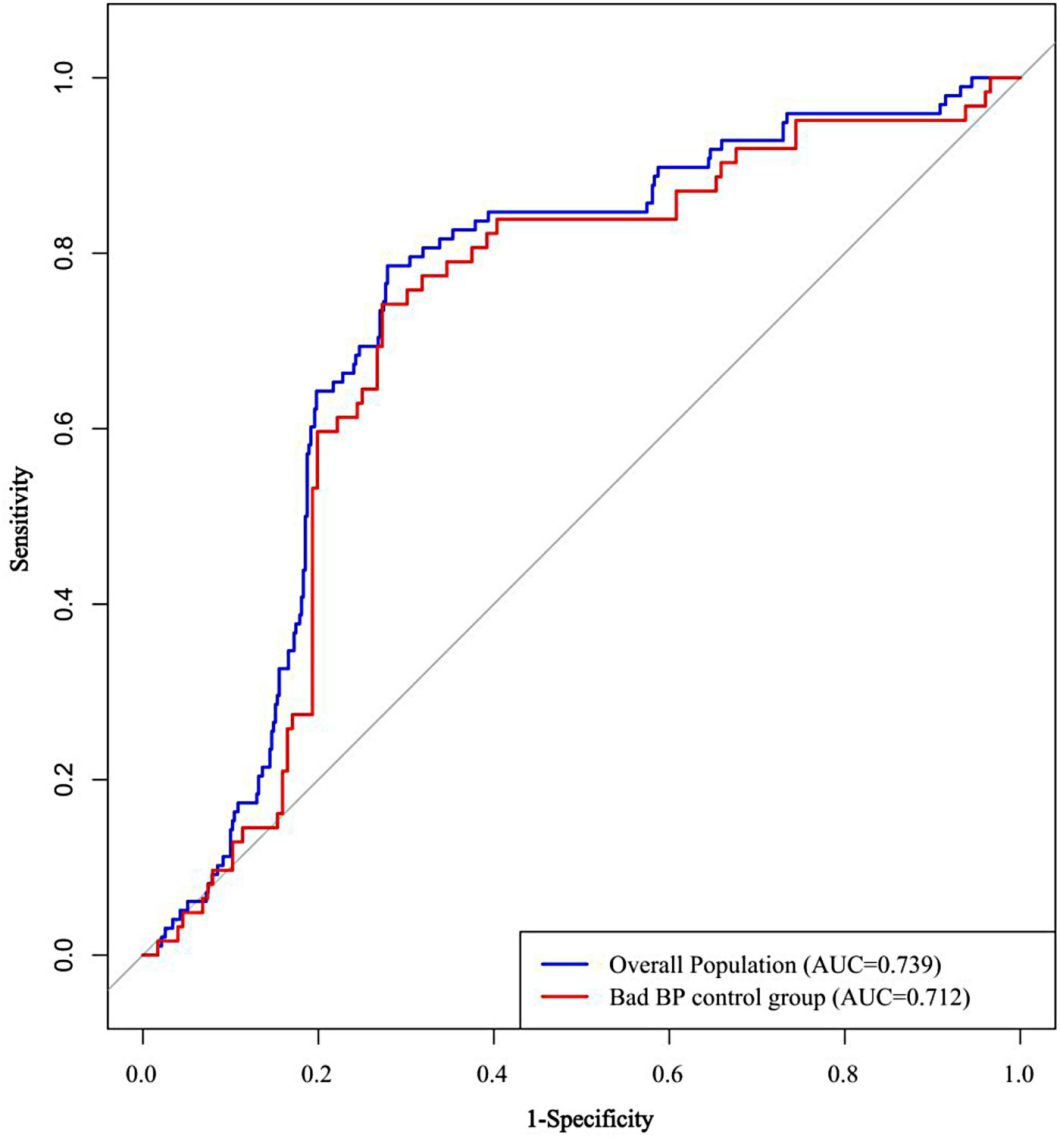
ROC curve analysis of SII for predicting AF recurrence.

ROC curve analysis was performed to evaluate the predictive utility of SII. It demonstrated significant predictive capacity for AF recurrence in the overall population (AUC = 0.739, 95% CI: 0.689–0.789), and maintained robust performance in the subgroup with poor blood pressure control (AUC = 0.712, 95% CI: 0.639–0.785) (Figure 3).

### 3.2 Predictive value of the SII and HTN control for AF recurrence

**Figure 4:**
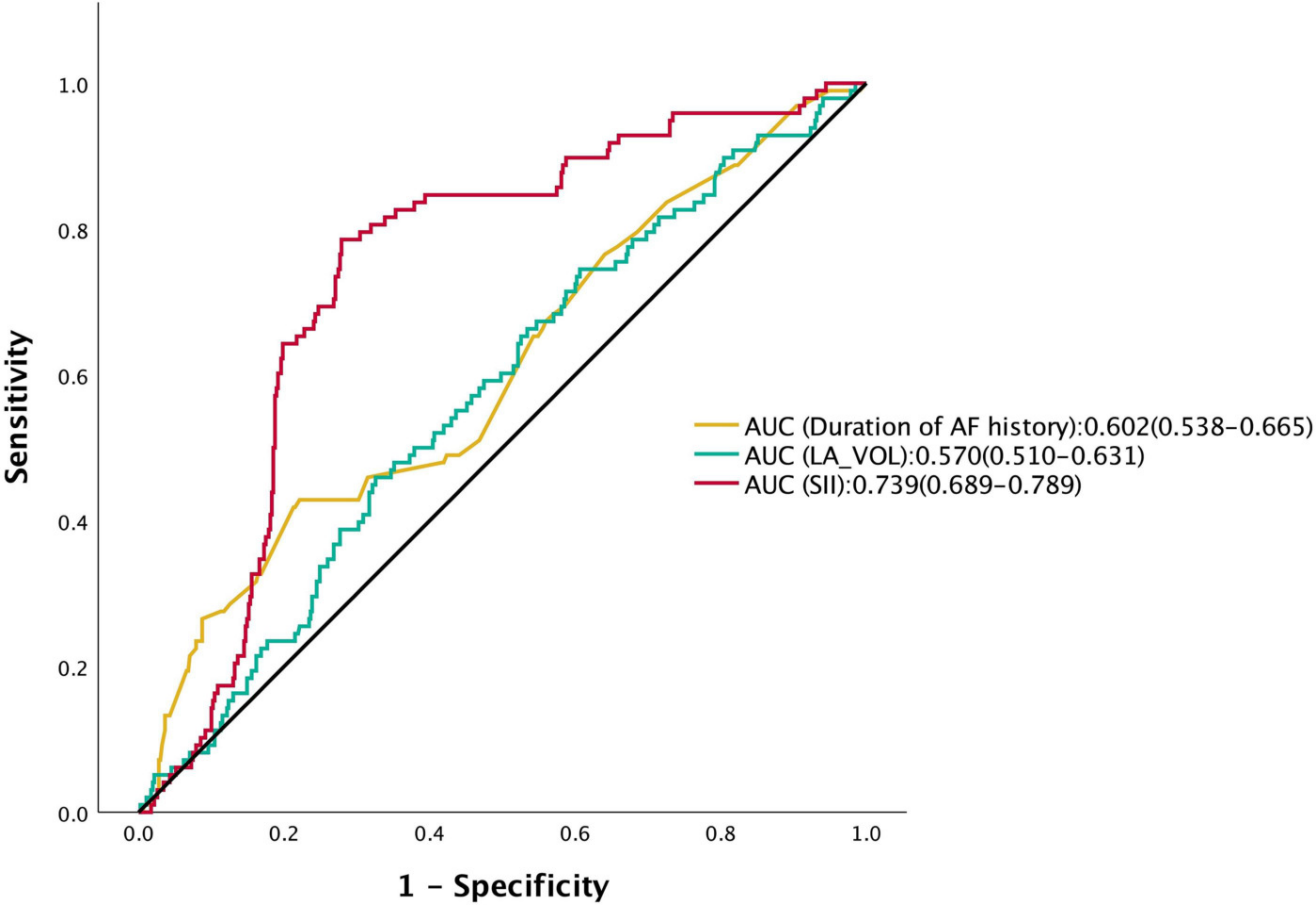
ROC curves comparing the predictive performance of pre-ablation SII, AF duration, and LA_VOL for AF recurrence after catheter ablation. AF, atrial fibrillation; AUC, area under the curve; CI, confidence interval; LA_VOL, Left atrial volume; ROC, receiver operating characteristic.

ROC curve analysis was conducted to evaluate the predictive performance of the SII, duration of AF, and left atrial volume for AF recurrence. The optimal cut-off value for SII was determined to be 554.63, yielding a sensitivity of 78.6% and a specificity of 72.1%, with an area under the curve (AUC) of 0.739 (95% CI: 0.689–0.789, *P* < 0.001) (Figure 4). In comparison, the AUCs for AF duration and left atrial volume were 0.602 (95% CI: 0.538–0.665) and 0.570 (95% CI: 0.510–0.631), respectively. According to the DeLong test, the AUC of SII was significantly greater than those of AF duration and left atrial volume (both *P* < 0.001), indicating superior predictive value.

**Figure 5:**
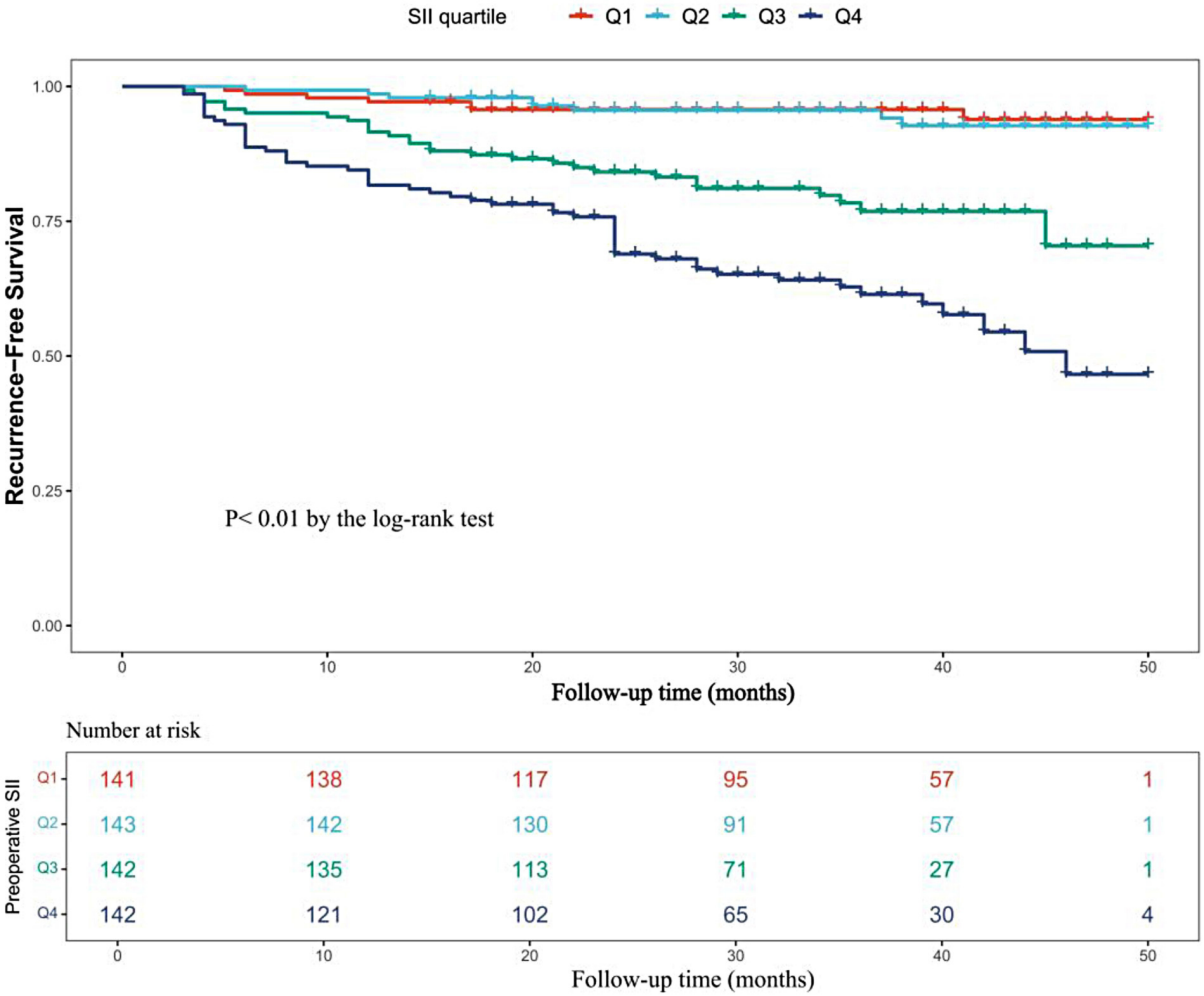
Kaplan–Meier survival curves for AF recurrence after catheter ablation, stratified by quartiles of the preoperative SII. Stratified by quartiles of SII. (Q1, SII ≤ 334.6; Q2, 334.6 < SII ≤ 463.08; Q3, 463.08 < SII ≤ 682.49; Q4, SII > 682.49). AF, atrial fibrillation; SII, systemic Immune-Inflammation Index.

Patients were stratified into four groups (Q1–Q4) based on SII quartile levels. Compared with the other subgroups, the Q4 group had significantly higher proportions of female patients, history of TIA, CHA₂DS₂-VASc scores, neutrophil counts, lymphocyte counts, platelet counts, and postoperative AF recurrence rates (all *P* < 0.05; Table 3). Kaplan–Meier survival analysis demonstrated a stepwise decline in AF recurrence-free survival across increasing SII quartiles (log-rank test, *P* < 0.001; Figure 5).

**Table 3:** Baseline characteristics of All patients stratified by quartiles of preoperative SII.

Parameters	Q1 (*N* = 142)	Q2 (*N* = 142)	Q3 (*N* = 142)	Q4 (*N* = 142)	*P*
Clinical characteristics					
Female, *n* (%)					
Age (years)	62.8 ± 8.63	62.3 ± 9.34	62.7 ± 9.46	62.4 ± 9.93	0.964
BMI (kg/m2)	25.4 ± 3.70	26.0 ± 3.20	26.3 ± 3.17	25.9 ± 3.27	0.167
Atrial fibrillation type					
Paroxysmal, *n* (%)	73 (51.4%)	68 (47.9%)	77 (54.2%)	66 (46.5%)	0.555
Persistent, *n* (%)	69 (48.6%)	74 (52.1%)	65 (45.8%)	76 (53.5%)	
Duration of AF history (months)	13 (2.0, 36.0)	13 (3.0, 48.0)	12 (2.3, 36.0)	12 (2.3, 60.0)	0.144
Current smoker, *n* (%)	28 (19.7%)	38 (26.8%)	45 (31.7%)	33 (23.2%)	0.118
Alcohol intake, *n* (%)	30 (21.1%)	35 (24.6%)	36 (25.4%)	29 (20.4%)	0.688
History of CAD, *n* (%)	56 (39.4%)	51 (35.9%)	55 (38.7%)	56 (39.4%)	0.918
Diabetes mellitus, *n* (%)	32 (22.5%)	34 (23.9%)	35 (24.6%)	32 (22.5%)	0.966
TIA, *n* (%)	27 (19.0%)	28 (19.7%)	48 (33.8%)	33 (23.2%)	0.012*
CHA2DS2-VASc score	3.40 ± 1.60	3.27 ± 1.57	3.40 ± 1.70	3.37 ± 1.72	0.012*
Echocardiographic					
Left ventricle diameter (mm)	47.4 ± 4.54	47.2 ± 3.95	47.7 ± 5.08	47.5 ± 5.72	0.864
Left atrial diameter (mm)	40.3 ± 6.30	40.1 ± 6.53	40.2 ± 6.30	40.8 ± 5.99	0.771
LVEF (%)	60.5 ± 6.64	61.4 ± 5.17	61.0 ± 6.78	59.7 ± 8.42	0.190
Left atrial volume(mm3)	156 ± 43.6	156 ± 53.7	155 ± 45.0	161 ± 47.2	0.743
Laboratory test					
RBC(×1012/L)	4.37 ± 0.499	4.42 ± 0.540	4.57 ± 0.546	4.39 ± 0.497	0.005*
PLTC(×109/L)	168 ± 36.8	197 ± 43.5	224 ± 46.8	249 ± 49.0	<0.001*
NEU(×109/L)	2.65 ± 0.675	3.75 ± 0.907	4.32 ± 0.940	5.19 ± 1.25	<0.001*
LYM(×109/L)	1.95 ± 0.473	1.82 ± 0.489	1.71 ± 0.438	1.47 ± 0.357	<0.001*
Alb(g/L)	41.1 ± 3.28	41.6 ± 3.95	42.0 ± 3.46	41.3 ± 3.85	0.180
TG (mmol/L)	1.49 ± 1.09	1.53 ± 0.798	1.51 ± 0.912	1.51 ± 1.69	0.996
LDL-C (mmol/L)	2.08 ± 0.734	2.09 ± 0.852	2.00 ± 0.722	2.16 ± 0.777	0.366
HDL-C (mmol/L)	1.10 ± 0.270	1.08 ± 0.267	1.04 ± 0.258	1.08 ± 0.254	0.399
eGFR (mL/min/1.73m2)	86.8 ± 15.0	88.1 ± 15.3	84.8 ± 17.3	84.4 ± 19.1	0.218
Cr (μmol/L)	72.5 ± 16.1	75.1 ± 25.7	79.4 ± 28.6	77.4 ± 21.2	0.074
BUA (μmol/L)	325 ± 94.6	327 ± 93.7	330 ± 99.9	326 ± 98.1	0.973
SII	229 ± 51.8	402 ± 35.1	561 ± 68.8	892 ± 269	<0.001*
Follow-up parameters					
Hypertension control statuses					
Bad blood pressure control, *n* (%)	53 (37.3%)	56 (39.4%)	64 (45.1%)	65 (45.8%)	0.386
Well blood pressure control, *n* (%)	89 (62.7%)	86 (60.6%)	78 (54.9%)	77 (54.2%)	
Recurrence, *n* (%)					
No	135 (95.1%)	134 (94.4%)	113 (79.6%)	88 (62.0%)	<0.001*
Yes	7 (4.9%)	8 (5.6%)	29 (20.4%)	54 (38.0%)	
Follow-up time (months)	34.1 ± 11.0	33.9 ± 10.5	29.2 ± 11.2	27.2 ± 13.1	<0.001*

Stratified by quartiles of SII. (Q1, SII ≤ 334.6; Q2, 334.6 < SII ≤ 463.08; Q3, 463.08 < SII ≤682.49; Q4, SII > 682.49) Data are expressed as mean ± standard deviation, median (interquartile spacing), or *n* (%).

AF, atrial fibrillation; Alb, albumin; BMI, body mass index; BUA, blood uric acid; CAD, coronary artery disease; Cr, creatinine; eGFR, estimated glomerular filtration rate; HDL-C, high-density lipoprotein cholesterol; LDL-C, low-density lipoprotein cholesterol; LYM, lymphocyte; LVEF, left ventricular ejection fraction; NEU, neutrophil; PLT, platelet; RBC, red blood cell; SII, systemic Immune-Inflammation Index; TG, triglycerides; TIA, Transient Ischemic Attack.

*P < 0.05.

Multivariate Cox regression analysis was conducted to examine the association between the SII, BP control status, and the risk of AF recurrence following catheter ablation. A stepwise modeling approach was adopted: Model 1 included no covariate adjustment; Model 2 adjusted for demographic variables (age and sex); and Model 3 further incorporated key clinical confounders, including BMI, AF type, AF duration, left atrial diameter、volume, and CHA₂DS₂-VASc score. Prior to multivariate analysis, the potential for multicollinearity among these variables—particularly between the CHA₂DS₂-VASc score and its individual components (age and sex)—was rigorously assessed using the Variance Inflation Factor (VIF). All included covariates exhibited VIF values well below the conservative threshold of 5 (maximum VIF = 2.184), confirming the statistical stability and validity of the final model. In all three models, SII consistently emerged as a significant independent predictor of recurrence [Model 3: hazard ratio [HR] per 100-unit increases in SII = 1.138; 95% confidence interval [CI]: 1.086–1.192; *P* < 0.001], indicating that each 100-unit rise in preoperative SII was associated with a 13.8% increased risk of AF recurrence.

**Figure 6:**
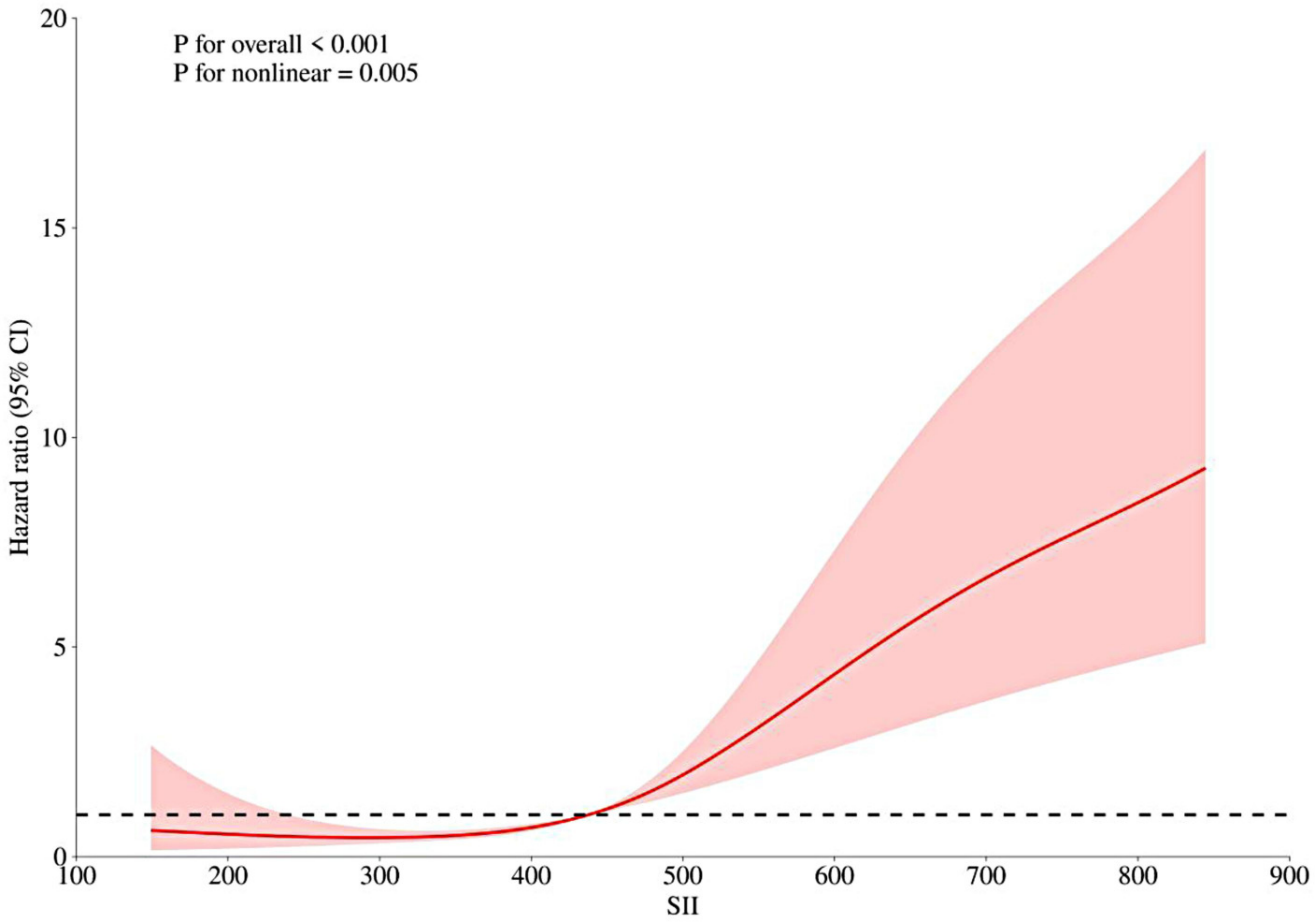
Nonlinear risk pattern between SII and AF recurrence. CI, confidence interval; RCS, restricted cubic spline; SII, systemic Immune-Inflammation Index.

When HTN control status was treated as a categorical variable, patients in the poorly controlled hypertension group exhibited a significantly elevated recurrence risk compared to those with good control (Model 3: HR = 2.405; 95% CI: 1.582–3.656; *P* < 0.001). Notably, patients with both poorly controlled HTN and SII in the highest quartile (Q4: >682.49) had a markedly increased risk of recurrence, with a more than fourfold elevation compared to the reference group (Model 3: HR = 4.061; 95% CI: 2.626–6.281; Table 4). Restricted cubic spline (RCS) analysis further revealed a significant nonlinear association between baseline SII and AF recurrence after full adjustment in Model 3 (*P* for non-linearity = 0.005; Figure 6).

**Table 4:** Combined effect of SII and HTN control Status on recurrence of AF.

Variables	Model 1		Model 2		Model 3	
	HR (95% CI)	*P*	HR (95% CI)	*P*	HR (95% CI)	*P*
SII (per 100-unit increase)	1.134 (1.085–1.185)	<.001	1.131 (1.083–1.181)	<.001	1.138 (1.086–1.192)	<.001
Poor_HTN						
No	(Reference)		(Reference)		(Reference)	
Yes	2.585 (1.714–3.899)	<.001	2.539 (1.679–3.840)	<.001	2.405 (1.582–3.656)	<.001
SII_Q4·Poor_HTN						
No	(Reference)		(Reference)		(Reference)	
Yes	4.351 (2.839–6.667)	<.001	4.282 (2.788–6.577)	<.001	4.061 (2.626–6.281)	<.001

The SII groups were stratified by the quartiles of the SII. (Q1, SII ≤ 334.6; Q2, 334.6 < SII ≤ 463.08; Q3, 463.08 < SII ≤ 682.49; Q4, SII > 682.49).

CI, confidence interval; HR, hazard ratio; HTN, hypertension; Poor_HTN, bad blood pressure control; SII, systemic Immune-Inflammation Index.

Model 1: unadjusted.

Model 2: adjust: age, gender.

Model 3: adjust: age, gender, AF-Type, BMI, CHA2DS2-VASc score, duration of AF history, LAD, LAVOL.

**Figure 7:**
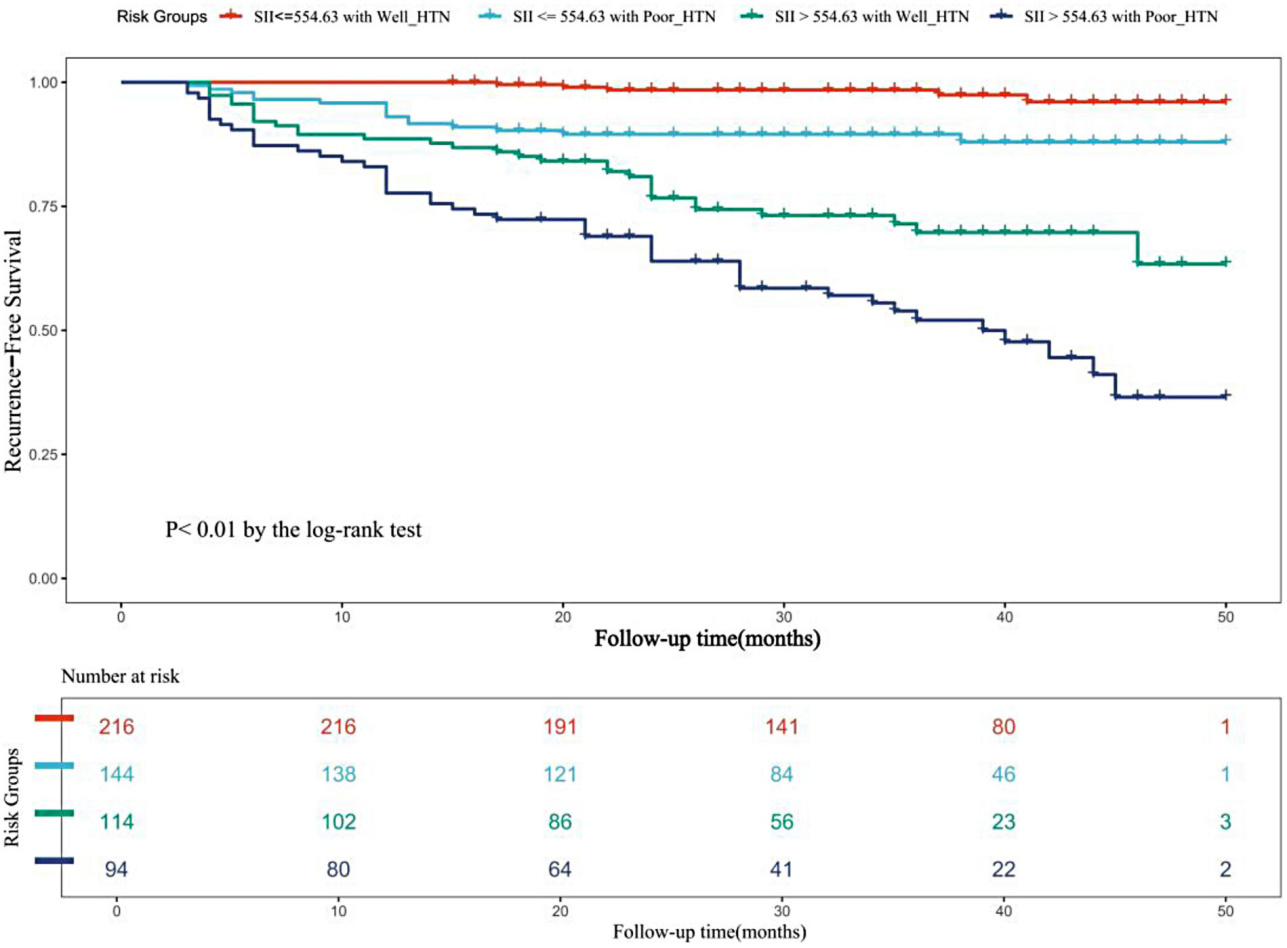
Cumulative incidence of AF recurrence stratified by HTN control Status and SII levels. AF, atrial fibrillation; HTN, hypertension; Poor_HTN, Bad blood pressure control; SII, systemic Immune-Inflammation Index; Well_HTN, well blood pressure control.

When all study participants were stratified into four groups based on HTN control status and SII levels (cut-off: 554.63), those with both poorly controlled HTN and elevated SII showed a significantly higher cumulative incidence of AF recurrence compared to the other subgroups (Figure 7).

### 3.3 Incremental predictive value of SII for AF recurrence after catheter ablation

Table 5 shows the incremental predictive value of the SII for AF recurrence. The baseline risk model had a C-statistic of 0.645 (95% CI: 0.586–0.704). After incorporating the SII into the model, there was a significant improvement in its predictive performance, with the C-statistic increasing to 0.755 (95% CI: 0.711–0.800, *P* < 0.001). To further ensure model stability, internal validation via 1,000-iteration bootstrap resampling was conducted, yielding a bias-corrected C-statistic of 0.764 for the final model. Additionally, the integrated discriminant improvement (IDI) was 0.033 (95% CI: 0.01–0.065, *P* < 0.001), and the net reclassification improvement (NRI) was 0.395 (95% CI: 0.164–0.511, *P* < 0.001), indicating that the inclusion of SII substantially enhanced the baseline model's predictive accuracy.

**Table 5:** Incremental predictive value of the SII for AF recurrence.

Model type	C-statistic (95% CI)	*P* value	IDI (95% CI)	*P* value	Continuous NRI (95% CI)	*P* value
Baseline risk model	0.645 (0.586–0.704)	Reference	Reference		Reference	
+SII	0.755 (0.711–0.800)	<0.001	0.033 (0.011–0.065)	<0.001	0.395 (0.164–0.511)	<0.001

Baseline risk model: age, gender, AF-Type, BMI, CHA2DS2-VASc score, duration of AF history, LAD, LAVOL.

AF, atrial fibrillation; CI, confidence interval; IDI, integrated discrimination improvement; NRI, net reclassification improvement; SII, systemic immune-inflammation index.

### 3.4 Subgroup analysis

**Figure 8:**
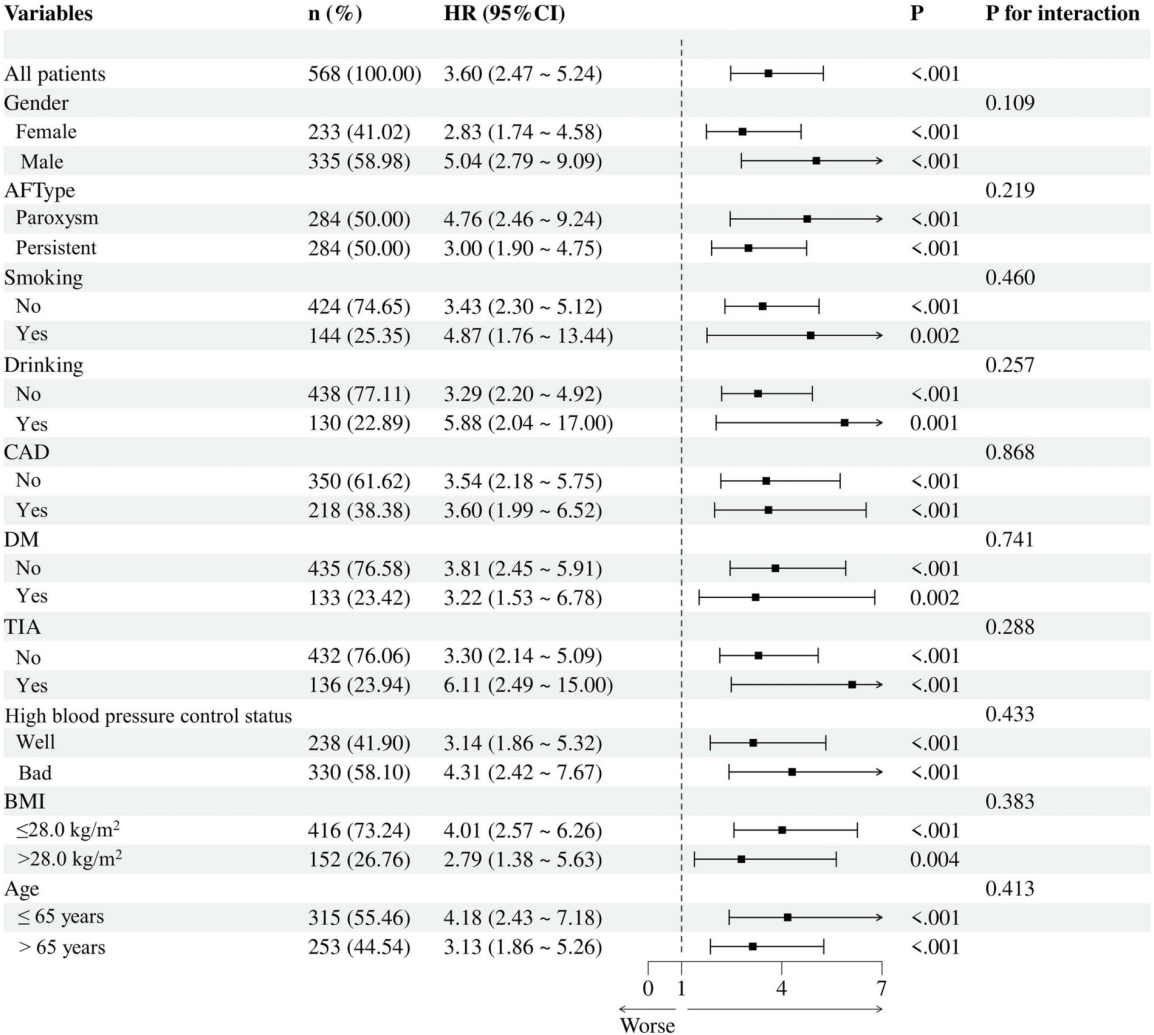
Subgroup analysis of the association between the SII and AF recurrence, stratified by clinical characteristics. AF, atrial fibrillation; BMI, body mass index; CAD, coronary atrial disease; DM, Diabetes mellitus; TIA, Transient Ischemic Attack.

Next, our subgroup analysis stratified based on sex, age, BMI, AF type, smoking status, alcohol consumption history, coronary heart disease, diabetes mellitus, transient ischemic attack, and hypertension control status is shown in Figure 8, where the SII index was associated with AF recurrence in all the different subgroups. However, the interaction test *P* values for all subgroups were >0.05, indicating that the main risk associations were consistent across subgroups with different clinical characteristics.

## 4 Discussion

In this study, we evaluated the predictive utility of the SII for AF recurrence following catheter ablation in a cohort of 568 hypertensive patients. We further examined the interplay effect of BP control by evaluating their joint predictive value, providing a more comprehensive risk stratification for AF recurrence. The main findings were as follows: (1) SII demonstrated robust predictive performance for AF recurrence (AUC = 0.739, 95% CI: 0.689–0.789; *P* < 0.001), outperforming traditional clinical markers such as AF duration (AUC =  0.602) and left atrial volume (AUC = 0.570), with the DeLong test confirming its staistical superiority (*P* < 0.001); (2) a significant joint effect was observed between elevated SII and poor blood pressure control; their combined presence was associated with a 4.061-fold increased risk of recurrence (HR = 4.061; *P* < 0.001); (3) incorporating SII into the baseline prediction model significantly enhanced its discriminative capacity, raising the C-statistic from 0.645 to 0.755; and (4) SII remained a significant predictor across all clinical subgroups, including stratifications by sex, age, BMI, AF type, comorbidities, and HTN control status. Collectively, these findings not only underscore the independent prognostic value of SII in cardiovascular disease but also highlight the critical role of BP management in mitigating post-ablation AF recurrence risk.

Recent evidence has established the SII as a critical hematologic marker reflecting the body's immunoinflammatory equilibrium (13). Its clinical utility in cardiovascular prognostication has been increasingly recognized. A large-scale multicenter meta-analysis involving 152,996 individuals demonstrated that elevated SII levels were robustly and positively correlated with the risk of major cardiovascular events, including acute coronary syndromes, various stroke subtypes, and peripheral vascular disease (8). Notably, a prospective cohort study reported that each unit increase in the natural logarithmic-transformed SII (lnSII) was associated with a 102% rise in cardiovascular mortality risk (*P* < 0.001) (14). After adjustment for potential confounders, individuals in the highest SII quartile remained at a significantly elevated risk of mortality (15). Consistent with these findings, our study revealed that patients in the highest SII quartile experienced a markedly increased rate of AF recurrence following ablation. Beyond observational associations, several studies have affirmed the prognostic relevance of SII in interventional cardiology settings. For instance, SII has demonstrated early predictive value for adverse clinical outcomes in patients with ST-segment elevation myocardial infarction (16) and has also been shown to effectively forecast arrhythmia recurrence following cryoballoon ablation (17). Importantly, a recent study focusing on patients with paroxysmal nonvalvular AF and comorbid hypertension reported that SII independently predicted AF recurrence after catheter ablation (12). Our current study extends this evidence base by confirming the independent predictive value of SII across all AF subtypes. Moreover, the prognostic utility of SII was particularly pronounced among patients with poorly controlled blood pressure, underscoring the clinical relevance of inflammation–hypertension interplay in AF recurrence risk stratification.

Hypertension serves not only as an independent risk factor for AF but also as a key determinant of post-ablation recurrence. Pathophysiological investigations have demonstrated that sustained elevations in arterial pressure contribute to left ventricular pressure overload, thereby facilitating the onset and perpetuation of AF through multiple mechanisms, including atrial fibrotic remodeling, ion channel dysregulation, and autonomic nervous system imbalance (5). Although preclinical studies suggest that aggressive antihypertensive therapy may mitigate the risk of AF recurrence (18), the influence of different blood pressure control statuses on long-term procedural outcomes remains incompletely elucidated. Importantly, a Mendelian randomization analysis using genetic instrumental variables confirmed a causal relationship between blood pressure indices and AF development, independent of conventional cardiovascular risk factors (19). Our cohort analysis supports this genetic evidence, showing that patients with uncontrolled hypertension had a significantly elevated risk of AF recurrence following ablation, with a hazard ratio of 2.40 (95% CI: 1.58–3.64, *P* < 0.001). This finding aligns with prior research and further underscores the pivotal role of postoperative blood pressure management in minimizing AF recurrence risk. Moreover, we observed that individuals in the poorly controlled blood pressure group exhibited higher rates of coronary artery disease (44.5% vs. 33.9%), diabetes mellitus (28.2% vs. 20.0%), and increased CHA₂DS₂-VASc scores (3.54 ± 1.70 vs. 3.23 ± 1.59). These findings suggest that suboptimal blood pressure control may exacerbate recurrence risk not solely through hemodynamic atrial remodeling, but also via a multifactorial pathological cascade. This includes accelerated coronary atherosclerosis, metabolic dysregulation, and an elevated propensity for thromboembolic events, collectively fostering a synergistic, multidimensional mechanism that drives AF recurrence beyond traditional structural remodeling pathways.

Several clinical studies have documented elevated neutrophil counts in individuals with hypertension (20, 21). Mechanistically, this elevation is thought to arise from trans endothelial migration facilitated by adhesion molecules such as β1-integrins, which promote inflammatory infiltration of the renal parenchyma and vascular wall (22–24). This process contributes to increased blood pressure through reactive oxygen species (ROS) production and the amplification of pro-inflammatory cascades, forming a self-perpetuating vicious cycle (22–24). In addition to neutrophils, platelets play a dual role in both inflammation and coagulation. One study demonstrated a causal link between platelet count and hypertension development (25), with hyperactivated platelets promoting thrombotic events (26). At the level of adaptive immunity, T lymphocytes are central regulators of immune activation in hypertension, and emerging evidence suggests that gut microbiota imbalances may influence blood pressure via T cell–mediated inflammatory signaling (27, 28). Moreover, a large genome-wide association study (GWAS) identified numerous single nucleotide polymorphisms (SNPs) associated with hypertension, many of which are involved in immune and inflammatory regulation pathways (29). These findings provide genetic support for the conceptual framework of this study, in which the SII functions not only as a marker of systemic inflammatory status, but also as a phenotypic proxy of genetic susceptibility, potentially modulating the risk of AF recurrence. Interestingly, although the present study revealed no statistically significant difference in SII values between patients with controlled and uncontrolled blood pressure (*P* = 0.0849), this may be attributed to the fact that SII reflects a transient immune-inflammatory state, while blood pressure control reflects a long-term, dynamic physiological adaptation. Hence, the two parameters may not always temporally align. Nonetheless, our multivariable regression analysis demonstrated a marked clinical synergy between high SII and poor blood pressure control. Patients exhibiting both factors faced a substantially heightened risk of AF recurrence (HR = 4.061, *P* < 0.001), far exceeding the risk associated with either factor alone. Although the effect size was modestly attenuated after model adjustments, the association remained highly significant and clinically meaningful, supporting the integration of SII and hypertension control into postoperative risk stratification frameworks. This finding not only reinforces the pathophysiological interplay between inflammation and hypertension, but also highlights the potential for precision medicine strategies that concurrently monitor systemic inflammation and optimize blood pressure control. Future investigations may explore targeted interventions addressing both pathways to enhance long-term outcomes in patients undergoing AF ablation.

This study has several limitations that merit consideration. First, the single-center retrospective design may introduce selection bias, despite our relatively large cohort. Second, although all patients followed standardized clinical protocols, the specific types and dosages of postoperative medications (e.g., antiarrhythmic and antihypertensive drugs) were not systematically recorded, which may influence the assessment of the SII's long-term predictive value. Third, as both blood samples and imaging were obtained upon admission, the potential impact of acute physiological stress and hemodynamic status (e.g., heart rhythm and fluid balance at the moment of imaging) on baseline SII and left atrial volume cannot be entirely ruled out. Although standardized fasting samples were collected the following morning to mitigate transient symptomatic interference, these fluctuations might still partially distort the representation of a stable baseline status. Fourth, a total of 62 patients (9.8% of the initial screening) were excluded due to missing laboratory data or loss to follow-up; the lack of complete records for these individuals precluded a formal baseline comparison, potentially introducing attrition bias. Fifth, conventional inflammatory biomarkers (e.g., CRP and ESR) were not included due to the retrospective nature of the study, limiting direct head-to-head comparisons between the SII and traditional markers. Finally, our follow-up relied on intermittent ECGs and Holter monitoring rather than continuous recording. Consequently, asymptomatic AF episodes may have been under-detected, potentially underestimating the true recurrence rate. Future studies using implantable recorders are needed to capture the total arrhythmia burden more precisely.

## 5 Conclude

This study is the first to evaluate the association between the SII and AF recurrence stratified by blood pressure control status. Our findings confirm that SII is an independent and superior predictor of AF recurrence following radiofrequency ablation, outperforming traditional markers such as AF duration and left atrial volume. Moreover, poor blood pressure control independently elevates recurrence risk, and a significant synergistic effect exists between elevated SII and uncontrolled hypertension. Joint assessment of SII and blood pressure control may enhance risk stratification and support tailored post-ablation management strategies. Future research should further investigate the potential benefits of anti-inflammatory therapies and intensive blood pressure management in reducing AF recurrence.

## Data Availability

The raw data supporting the conclusions of this article will be made available by the authors, without undue reservation.
